# Rush Medical College

**Published:** 1849-07

**Authors:** 


### ARTICLE IV.

### RUSH MEDICAL COLLEGE.

The friends of this Institution will be gratified to learn that the vacant chairs have been filled by the appointment of gentlemen of high character, and extensive acquirements; both of whom are well known to the profession throughout the whole country.

Dr. Thomas Spencer, elected to the chair of the Principles and Practice of Medicine, lately filled the same chair in Geneva Medical College in Western New York, of which he may be considered the founder, and which ranked among the first Institutions in this country during his connection with it.

He is extensively known as an able writer and as a skilful practitioner of medicine.

He has enjoyed the advantage of a visit to the great Hospitals of Europe. Also of an extensive practice for years in a malarious district of country, so that he is familiar with the diseases of the West.

Dr. N. S. Davis, who is appointed Professor of Physiology and Pathology, is the present able Editor of the "Annalist and Record of Practical Medicine in the City of N. Y." He is the author of several prize essays and valuable papers on medical subjects, which have been published.

He gave a highly satisfactory course of lectures in the College of Physicians and Surgeons of N. Y., last spring. But he is especially distinguished by his efforts at medical reform and improvement; he being the originator of the plan of, and an active agent in putting into operation, the National Medical Association, which has been crowned with the happiest results.

It is understood that both these gentlemen will make their future residence in the West.

